# Clinical Impact of Using Real-Time Image-Processing Algorithms (Comb Removal and Image Sharpening) in Dacryoendoscopic Surgery

**DOI:** 10.3390/jcm15051951

**Published:** 2026-03-04

**Authors:** Kuniharu Tasaki, Sujin Hoshi, Takahiro Hiraoka, Tetsuro Oshika

**Affiliations:** Department of Ophthalmology, Institute of Medicine, University of Tsukuba, Ibaraki 305-8575, Japan; hoshisujin@md.tsukuba.ac.jp (S.H.); thiraoka@md.tsukuba.ac.jp (T.H.); oshika@eye.ac (T.O.)

**Keywords:** dacryoendoscopy, lacrimal duct obstruction, image-processing algorithm, image-sharpening algorithm, comb-removal algorithm

## Abstract

**Background/Objectives:** Lacrimal passage intubation is commonly used to treat lacrimal duct obstruction. However, conventional dacryoendoscopes, which are limited by their low resolution and comb-structure artifacts, pose challenges for visualization. Two novel image processing algorithms—comb removal and image sharpening—have been developed to enhance visibility, and this study aimed to evaluate the clinical effects of these algorithms on the outcomes of dacryoendoscopic surgery. **Methods:** A retrospective study was conducted on 121 sides of 84 patients (mean age, 72.3 ± 10.5 years) who had undergone lacrimal passage intubation. The patients were categorized into comb-removal and image-sharpening groups according to the algorithm used during the procedure. The clinical parameters of pain score, endoscopy duration, irrigation fluid volume, and irrigation fluid flow rate were compared between the groups using the linear mixed-effects model, and recurrence rates were evaluated using Kaplan–Meier analysis. **Results:** The image-sharpening algorithm was associated with a significant reduction in irrigation fluid usage (β = −1.34 mL, SE = 0.52, *p* = 0.012), with the pain score (β = −1.71, SE = 0.93, *p* = 0.069) and endoscopy duration (β = −0.50 min, SE = 0.39, *p* = 0.199) also showing reduction trends, but these did not reach statistical significance. The comb-removal algorithm showed no significant associations with any evaluated outcome. Recurrence rates were similar between the groups. **Conclusions:** Real-time image sharpening was associated with improved procedural efficiency during dacryoendoscopic surgery, while clinical outcomes showed favorable trends that did not reach statistical significance. These findings suggest a potential supportive role of these algorithms in intraoperative handling; however, whether this translates into clinically meaningful benefits requires further investigation.

## 1. Introduction

Dacryocystorhinostomy and lacrimal passage intubation (LPI) are the primary treatment options for lacrimal duct obstruction. In the past, LPI was performed indiscriminately, with a recurrence rate of 16.5–49.3% [1,2,3,4], resulting in suboptimal outcomes. However, dacryoendoscopy has significantly improved treatment success, reducing the recurrence rate to 11.3–24.1% [5,6,7].

LPI is often performed under local anesthesia, during which irrigation fluid flows into the pharynx or patients frequently experience discomfort because of pain. Currently, dacryoendoscopes with an outer diameter of 0.9 mm are commonly used; however, challenges remain, such as the low resolution (approximately 10,000 pixels) and visual impairments caused by honeycomb-structure artifacts due to the boundaries of the fiber bundles. Enhancing visual quality can improve procedural accuracy and reduce patient burden.

**Figure 1:**
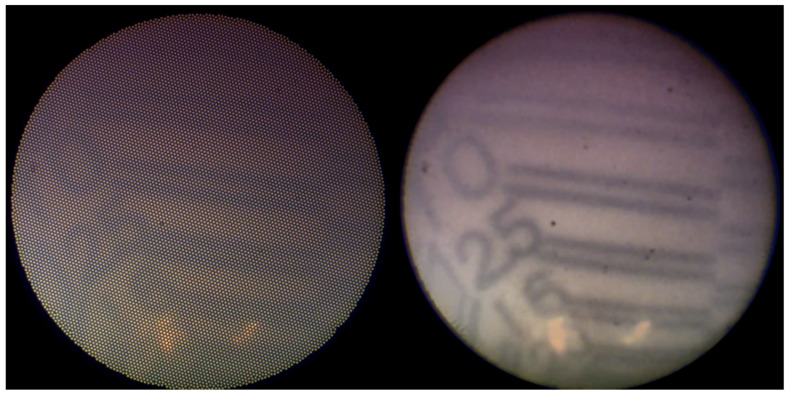
Observation of a visual target in a suspension using dacryoendoscopy. The (**left**) panel shows the unprocessed image, and the (**right**) panel shows the processed image. The comb-removal algorithm eliminates the honeycomb pattern due to the fiber bundle boundaries of the endoscope, while the image-sharpening algorithm enhances the contrast of the visual target.

Emerging technologies, such as comb-removal and image-sharpening algorithms, can potentially address these challenges. These techniques have improved visibility in dacryoendoscopy and ophthalmic surgery [8,9]. However, their clinical utility remains unclear (Figure 1).

Because dacryoendoscopic LPI is performed within a narrow and complex luminal structure, the surgeon must rely heavily on endoscopic visualization to identify the true passage and avoid false routes. In such confined environments, visualization quality directly influences manipulation strategy and procedural efficiency rather than acting solely as an optical improvement.

Evidence from other endoscopic disciplines supports this concept. In gastrointestinal endoscopy, narrow-band imaging improves mucosal contrast but does not consistently improve detection outcomes, suggesting that its primary role is enhancing operator performance rather than direct therapeutic impact [10]. Similarly, in respiratory endoscopy, image-enhanced bronchoscopy facilitates the recognition of abnormal microvascular patterns without uniformly altering patient-related procedural outcomes [11].

Therefore, improving visualization during dacryoendoscopic LPI may primarily influence procedural handling, potentially reducing unnecessary manipulation and patient discomfort. The present study aimed to evaluate whether real-time image-processing algorithms improve procedural performance during dacryoendoscopic LPI using linear mixed-effects modeling to account for clinical heterogeneity.

## 2. Materials and Methods

This study adhered to the principles of the Declaration of Helsinki and was approved by the Ethics Committee of the Faculty of Medicine, University of Tsukuba (Approval No. R03-179), as an observational study.

All surgical procedures were performed as part of routine clinical care, and written informed consent for the procedures was obtained from all patients prior to treatment. The clinical data used in this study were retrospectively analyzed and were not collected for research purposes. The study hypothesis and analysis plan were defined data collection.

Because this study used anonymized clinical data, the requirement for additional informed consent for research participation was waived, and an opt-out consent process was implemented.

### 2.1. Participants

We included consecutive patients who underwent LPI at University of Tsukuba Hospital between 1 April 2022 and 31 May 2023. Patients with severe obstructions that could not be treated using standard techniques, iatrogenic obstructions due to chemotherapy, and acute dacryocystitis or dacryolithiasis were excluded.

### 2.2. Surgical Technique and Evaluation Metrics

All surgeries were performed by three experienced surgeons (K.T., H.M. and T.H.). Before data collection, all surgeons completed an adaptation phase to the image-processing algorithms, each performing more than 20 procedures. As previously described, LPI was performed using a combination of sheath-guided endoscopic probing and sheath-guided intubation [9]. The anesthesia protocol involved infratrochlear nerve block with 1% lidocaine and the irrigation of the canalicular system with a 4% lidocaine solution, followed by punctal dilation. The dacryoendoscope (LAC-06NZ-HS; MACHIDA Endoscope Co., Ltd., Chiba, Japan) was covered with a sheath prepared using an 18-gauge plastic cannula (SurFlash^®^ Polyurethane IV Catheters; Terumo Corporation, Tokyo, Japan). The sheath-equipped dacryoendoscope was inserted into the punctum, and sheath-guided endoscopic probing was performed by probing the obstructed area. Saline was continuously irrigated using a foot pedal (Lacri-Flush^®^; MACHIDA Endoscope Co., Ltd., Chiba, Japan) throughout the procedure. The sheath was temporarily retained for use as a guide during sheath-guided intubation, and an 11 cm long polyurethane lacrimal duct stent tube (LACRIFAST^®^; KANEKA Corporation, Tokyo, Japan) was inserted through the nasal cavity.

Pain was assessed immediately after the procedure using a 10-point visual analog scale (VAS; 0 = no pain and 10 = worst imaginable pain) based on the patient’s self-reported perception. Endoscopy duration was retrospectively determined by reviewing the recorded endoscopic videos and summing only the periods during which the endoscope was actively manipulated within the lacrimal passage, excluding extra-luminal handling time. The total irrigation fluid volume used during the procedure was determined by nursing staff from the remaining volume in the syringe after completing irrigation. The irrigation fluid flow rate per unit time (mL/min) was calculated by dividing the total irrigation volume by the endoscopy duration. The recurrence of lacrimal duct obstruction was defined as failure to obtain patency upon lacrimal irrigation testing during follow-up. Data regarding recurrence were collected from medical records.

### 2.3. Image Processing

During dacryoendoscopy, the video output from the fiberscope was routed to an external real-time image-processing unit and displayed on the monitor without perceptible latency (approximately 4 ms). This experiment evaluated a fiber-pattern suppression process (WipeFiber^®^, WF) and a local dynamic range optimization process (Medical Image Enhancer, MIEr^®^). An additional condition in which both processes were applied simultaneously was also analyzed.

Because fiberscope images are composed of multiple optical fiber elements, a fixed mesh-like pattern may appear and interfere with the visualization of the luminal wall and obstruction margin. WF suppresses this structural pattern component and generates a visually continuous image, thereby reducing structural noise.

MIEr analyzes luminance distribution in very small regions and optimizes the local dynamic range to improve overall image visibility. By independently correcting high- and low-contrast areas, it preserves information in darker regions while preventing saturation in brighter regions, enabling the uniform visualization of the duct wall and obstruction boundary. An auxiliary sharpening step further enhances boundary information and improves the detectability of mucosal irregularities and stenotic segments. This process does not involve pixel interpolation or artificial texture generation.

In the combined condition, both processes were applied simultaneously.

To avoid variability in observation conditions, the image-processing settings were fixed before the procedure and were not adjusted by the surgeon during surgery. All procedures for each patient were performed on the same day, and the surgeon used the same combination of image-processing algorithms throughout the procedure without modifying the settings. Accordingly, in bilateral cases, both sides were treated using the same combination of algorithms.

To reduce temporal confounding while maintaining routine clinical workflow, the implementation of WF and MIEr was rotated according to a pre-specified calendar schedule (WF in two-week blocks and MIEr in one-week blocks). The schedule was determined independently of patient characteristics and was not modified based on case complexity or intraoperative findings.

### 2.4. Statistical Analysis

Continuous outcomes (pain score, endoscopy duration, irrigation fluid volume, and irrigation fluid flow rate) were analyzed using linear mixed-effects models (LMMs) to account for within-patient correlation in bilateral cases. A random intercept for the patient was included. The use of the comb-removal algorithm (WF) and the image-sharpening algorithm (MIEr) was modeled as fixed effect, and sex, age, laterality, obstruction type, and surgeon were included as additional fixed effects. The models were fitted using restricted maximum likelihood (REML), and denominator degrees of freedom were estimated using the Satterthwaite approximation. Adjusted marginal means (95% confidence intervals) were derived from the fitted models.

Recurrence-free survival was evaluated using Kaplan–Meier curves and compared using the log-rank test. Statistical significance was defined as a two-sided *p* < 0.05. All analyses were performed using IBM SPSS Statistics (Version 29.0; IBM Corp., Armonk, NY, USA).

## 3. Results

This study involved 121 sides of 84 patients (23 males and 61 females; mean age, 72.3 ± 10.5 years) diagnosed with lacrimal duct obstruction. The distribution of the disease type by site was as follows: punctal obstruction, 9 sides (7.4%); canalicular obstruction, 13 sides (10.7%); nasolacrimal duct obstruction, 59 sides (48.8%); common canalicular and nasolacrimal duct obstruction, 26 sides (21.5%); and lacrimal duct stenosis, 14 sides (11.6%).

The comb-removal and image-sharpening algorithms were applied to 50 and 71 sides, respectively. In a total of 37 sides, both algorithms were used; however, in another 37, neither was.

Linear mixed-effects models adjusted for age, sex, laterality, surgeon, and disease type were used to evaluate the association between real-time image-processing algorithms and clinical outcomes. Denominator degrees of freedom were estimated using the Satterthwaite approximation. Interaction between WF and MIEr was tested, but as it was not significant, it was excluded from the final model. All adjusted estimates are summarized in Table 1.

### 3.1. Pain Score

The comb-removal algorithm was not associated with intraoperative pain (β = −0.68, SE = 0.70, *p* = 0.331), whose adjusted mean scores were 3.48 (95% CI, 1.42–5.55) without the comb-removal algorithm and 2.80 (95% CI, 0.57–5.03) with the comb-removal algorithm.

The image-sharpening algorithm showed a reduction trend in pain score after multivariable adjustment (β = −1.71, SE = 0.93, *p* = 0.069). The adjusted mean pain scores were 3.99 (95% CI, 1.44–6.56) without the image-sharpening algorithm and 2.28 (95% CI, 0.43–4.14) with the image-sharpening algorithm.

### 3.2. Irrigation Fluid Volume

The comb-removal algorithm showed no significant effect on irrigation fluid volume (β = +0.14 mL, SE = 0.39, *p* = 0.724), whose adjusted mean values were 2.64 mL (95% CI, 1.48–3.80) without the comb-removal algorithm and 2.78 mL (95% CI, 1.53–4.02) with the comb-removal algorithm.

In contrast, the image-sharpening algorithm significantly reduced irrigation fluid usage (β = −1.34 mL, SE = 0.52, *p* = 0.012). The adjusted mean irrigation volume values were 3.37 mL (95% CI, 1.94–4.81) without the image-sharpening algorithm and 2.04 mL (95% CI, 1.00–3.08) with the image-sharpening algorithm.

### 3.3. Endoscopy Duration

Neither algorithm significantly affected endoscopy duration. For the comb-removal algorithm, the adjusted difference was β = +0.10 min (SE = 0.30, *p* = 0.744), with adjusted mean values of 2.75 min (95% CI, 1.89–3.61) without the comb-removal algorithm and 2.84 min (95% CI, 1.91–3.77) with the comb-removal algorithm.

For the image-sharpening algorithm, the adjusted difference was β = −0.50 min (SE = 0.39, *p* = 0.199), with adjusted mean values of 3.05 min (95% CI, 1.98–4.11) without the image-sharpening algorithm and 2.54 min (95% CI, 1.77–3.32) with the image-sharpening algorithm, indicating a reduction trend.

### 3.4. Irrigation Fluid Flow Rate

No significant associations were observed between either algorithm and irrigation fluid flow rate. For the comb-removal algorithm, the adjusted mean flow rates were 1.32 mL/min (95% CI, −0.37 to 3.02) without the comb-removal algorithm and 1.79 mL/min (95% CI, 0.61 to 2.97) with the comb-removal algorithm (β = +0.47, SE = 0.40, *p* = 0.249).

The image-sharpening algorithm showed a reduction trend in irrigation fluid flow rate after multivariable adjustment (β = −0.85, SE = 0.42, *p* = 0.168). The adjusted mean irrigation fluid flow rates were 1.06 mL/min (95% CI, 0.22–1.89) without the image-sharpening algorithm and 0.21 mL/min (95% CI, −0.62–1.05) with the image-sharpening algorithm.

### 3.5. Recurrence Rates

**Figure 2:**
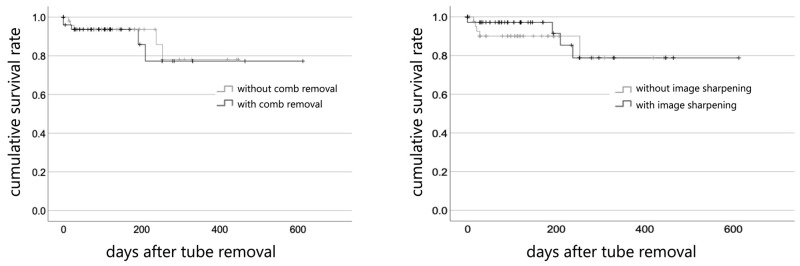
Comparison of Kaplan–Meier curves relative to the groups where the comb-removal algorithm (**Left**) and image-sharpening algorithm (**Right**) were and were not used. No significant differences in recurrence rates were observed for either the comb-removal algorithm (*p* = 0.68, log-rank test) or the image-sharpening algorithm (*p* = 0.61, log-rank test).

In the group where the comb-removal algorithm was not used (n = 71), recurrence was observed in 5 (7.0%) of 71 cases, compared with 5 (10.0%) of 50 cases in the group where the algorithm was employed (n = 50), showing no significant differences (*p* = 0.68). In the group where the image-sharpening algorithm was not adopted (n = 50), recurrence was observed in 5 (10.0%) of 50 cases, compared with 5 (7.0%) of 71 cases in the group where the algorithm was used (n = 71), also showing no significant differences (*p* = 0.61) (Figure 2).

## 4. Discussion

In the present study, the image-sharpening algorithm was associated with reductions in pain score and endoscopy duration after multivariable adjustment, whereas irrigation fluid volume exhibited a statistically significant reduction. These findings indicate that improved image clarity primarily enhances procedural handling, with the most robust measurable effect having been observed in irrigation efficiency.

This interpretation aligns with evidence from other endoscopic fields. In gastrointestinal endoscopy, narrow-band imaging enhances mucosal and vascular contrast; nevertheless, prospective comparative studies have shown that such enhancement does not consistently improve polyp detection compared with conventional colonoscopy [10]. This suggests that enhanced visibility alone does not automatically translate into superior clinical endpoints but instead improves inspection behavior and observational precision. Likewise, in respiratory endoscopy, NBI bronchoscopy increases the recognition of abnormal mucosal microvasculature and improves early lesion detection [11], yet its principal contribution lies in visual discrimination and navigational assistance rather than direct improvement in patient comfort or procedural duration.

**Figure 3:**
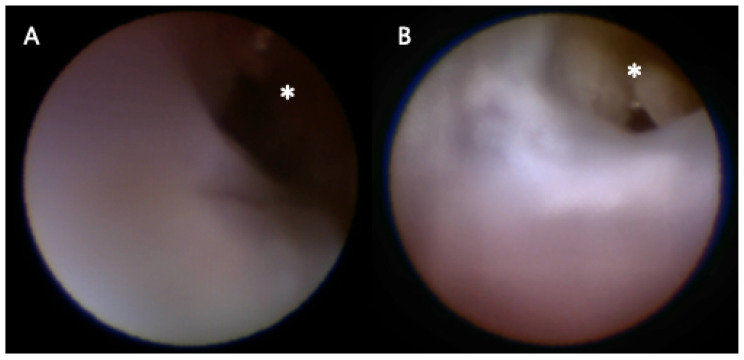
Image of the nasolacrimal duct without (**A**) and with (**B**) the application of the image-sharpening algorithm. The image-sharpening algorithm significantly improved endoscopic visibility, particularly in the distal region (*).

Because dacryoendoscopy is performed within a narrow and tortuous lumen, visualization quality directly influences procedural handling. As illustrated in Figure 3, the image-sharpening algorithm enhanced visualization of the distal lumen, which may facilitate smoother instrument advancement and more targeted irrigation. In this context, the significant reduction in irrigation fluid usage together with the tendencies toward lower pain scores and shorter endoscopy duration suggests improved procedural efficiency during dacryoendoscopic surgery.

In contrast, the comb-removal algorithm did not demonstrate measurable benefit across the evaluated outcomes. This suggests that enhancing structural contrast—improving edge and texture definition—may be more functionally relevant for surgical navigation in confined luminal systems than the cosmetic removal of fiber-pattern artifacts. In small-lumen endoscopy, visual information that enhances spatial orientation appears to be more critical than esthetic image refinement.

Overall, real-time image sharpening improved operational efficiency in dacryoendoscopic surgery and was accompanied by favorable trends in patient discomfort and manipulation time. These findings are consistent with the possibility that image-processing technologies in endoscopy influence procedural performance; however, whether they also translate into clinically meaningful therapeutic benefits remains to be determined.

This study has several limitations. First, it was a retrospective single-center analysis, and the allocation of image-processing modes was not randomized. Although statistical adjustment using a linear mixed-effects model was performed, residual confounding could not be entirely excluded. Second, the number of independent subject-level observations may have limited statistical power for subjective outcomes such as pain score. This limited power may explain why the reduction trend did not reach statistical significance despite consistent directional effects. Third, surgeons were able to recognize signs of the activation of the image-sharpening system on the monitor, which could have theoretically influenced operator behavior. Awareness of enhanced visualization might alter manipulation strategy, procedure pacing, or irrigation use. To address this concern, we evaluated both objective efficiency metrics (irrigation fluid volume and flow rate per unit time) and a patient-reported outcome (pain score). The concordance of findings across these independent domains supports the internal consistency of the results and reduces the likelihood that the observed effects are solely attributable to systematic behavioral bias. Furthermore, the absence of measurable effects with the comb-removal algorithm provides indirect support for the efficacy of the image-sharpening algorithm.

Although the image-sharpening algorithm improved several clinical parameters, neither algorithm was associated with a difference in recurrence rates. The recurrence rate was low in both groups (approximately 7–10%), resulting in a limited number of events and likely insufficient statistical power to detect differences between the two image-processing modes. Therefore, the lack of significant differences in recurrence should be interpreted with caution. Because this study was not primarily designed to evaluate recurrence, larger studies are needed to determine whether image processing influences long-term outcomes. In addition, future research should examine other potential determinants of recurrence, such as obstruction severity and surgical technique.

## 5. Conclusions

The image-sharpening algorithm was associated with improved procedural efficiency during dacryoendoscopic surgery, as demonstrated by a significant reduction in irrigation fluid usage. Pain score and procedure duration showed favorable reduction trends but did not reach statistical significance. These findings suggest that real-time image processing may support intraoperative handling, but its impact on clinically meaningful outcomes requires further investigation. Given the relatively limited sample size, these results should be interpreted with appropriate caution. To our knowledge, this is the first study to demonstrate the practical impact of real-time image processing on operative performance in dacryoendoscopic surgery.

## Figures and Tables

**Table 1:** Adjusted comparison of clinical outcomes. Values are adjusted marginal means (95% confidence intervals) derived from linear mixed-effects models fitted with REML and Satterthwaite degrees of freedom. A random intercept for patient was included to account for bilateral cases. Fixed effects included algorithm use (WF and/or MIEr), age, sex, laterality, obstruction type, and surgeon. β represents the adjusted difference (with vs. without algorithm use), and 95% confidence intervals were calculated using the Wald method.

Outcome	Algorithm	Without Mean (95% CI)	With Mean (95% CI)	β (SE)	95% CI	*p*
Pain Score	WF	3.48 (1.42–5.55)	2.80 (0.57–5.03)	−0.68 (0.70)	−2.05–0.69	0.331
	MIEr	3.99 (1.44–6.56)	2.28 (0.43–4.14)	−1.71 (0.93)	−3.53–0.11	0.069
Irrigation Fluid Volume	WF	2.64 (1.48–3.80)	2.78 (1.53–4.02)	+0.14 (0.39)	−0.62–0.90	0.724
	MIEr	3.37 (1.94–4.81)	2.04 (1.00–3.08)	−1.34 (0.52)	−2.36–−0.32	0.012
Endoscopy Duration	WF	2.75 (1.89–3.61)	2.84 (1.91–3.77)	+0.10 (0.29)	−0.49–0.69	0.744
	MIEr	3.05 (1.98–4.11)	2.54 (1.77–3.32)	−0.50 (0.39)	−1.27–0.27	0.199
Irrigation Fluid Flow Rate	WF	1.32 (−0.37–3.02)	1.79 (0.61–2.97)	+0.47 (0.40)	−0.33–1.26	0.249
	MIEr	1.06 (0.22–1.89)	0.21 (−0.62–1.05)	−0.85 (0.42)	−1.67–−0.03	0.168

## Data Availability

The datasets generated during and/or analyzed during the current study are available from the corresponding author upon reasonable request.

## Abbreviations

The following abbreviations are used in this manuscript:

LMM	Linear mixed-effects model
LPI	Lacrimal passage intubation
